# Efficacy of a bacterial bedding conditioner in the reduction of footpad lesions in broilers

**DOI:** 10.3389/fvets.2025.1661293

**Published:** 2025-11-18

**Authors:** Livio Galosi, Lucia Biagini, Anna-Rita Attili, Adolfo Maria Tambella, Giacomo Rossi, Giorgio Provolo, Roberto Falconi, Giulia Castiglione, Alessandra Roncarati

**Affiliations:** 1 School of Biosciences and Veterinary Medicine, University of Camerino, Matelica, MC, Italy; 2 Department of Agricultural and Environmental Sciences, University of Milan, Milan, Italy

**Keywords:** bacterial bedding conditioner, broilers, farming management, footpad lesions, litter

## Abstract

Footpad dermatitis is a multifactorial condition that affects broiler welfare. We aimed to evaluate whether a bacterial compound sprinkled on the litter could have a positive effect on the formation of footpad lesions (FPL), a trial was conducted under productive conditions. A total of 89,200 Ross308 chickens (39 ± 3 g) were housed in two sheds (C, control; T, treated). Females were housed in the first part of each shed, and males in the second and third part. A bacterial bedding conditioner was applied in T, while no treatments were carried out in C. Females were slaughtered at 36d (T: 1528 ± 195 g; C: 1562 ± 188 g) and males utilized the whole space until 43d (T: 2696 ± 296 g; C: 2737 ± 364 g). At 20d and before capture, 30 birds living in each part of the shed were randomly selected and, from both pads, the perimeter shape of each lesion was recorded, and the FPL area was measured. Litter was sampled at the same timepoints, for bacteriological and mycological culture, and chemical analysis. At slaughtering, 12 legs for females and 12 for males, both C and T, were randomly selected for histological examination. At day 20, FPL were not observed in birds. At 36 days, lesions measured in T (0.57 ± 0.08 cm^2^) were significantly lower than in C (1.47 ± 0.14 cm^2^; *p* < 0.0001). At 44d, lesions measured in T (0.65 ± 0.18 cm^2^) were significantly lower than in C (1.76 ± 0.34 cm^2^; *p* = 0.049). In litter collected in T, a significant reduction was observed for Gram negative bacteria (*p* = 0.0015) and *Staphylococcus* spp. (*p* = 0.0386), particularly in the second part of the shed (*p* = 0.0098, *p* = 0.0131 respectively). Regarding FPL, birds farmed in T showed a total histological score significantly lower than in C (*p* = 0.0002), more specifically for males (*p* < 0.0001). The use of the compound positively affected width and severity of FPL, supported by bacteriological analysis that evidenced a reduction of the total charge of bacteria commonly involved in the disease.

## Introduction

Considering the rising of consumer awareness of animal welfare in intensive farming, it is becoming increasingly urgent to find innovative solutions to problems that can affect the quality of life of animals. The European Directive 2007/43/CE “laying down minimum rules for the protection of chicken kept for meat production” introduced improvements for animal welfare, requiring producers to implement technical and management measures (1). As part of the slaughterhouse checks, the official veterinarian must identify any indicators of poor welfare, among which, in first place, the legislator places the “contact dermatitis.” A report on the application of the above-mentioned Directive (2) claims that 8 Member States have the obligation to record cases of footpad dermatitis (FPD) in accordance with national legislation. Fifteen of these states have established a link between this legal obligation and the implementation of targeted actions. FPD is a condition that causes necrotic lesions on the plantar surface of growing broilers, affecting their welfare and representing a risk factor, since it is one of the main entry points for pathogenic microorganisms (3). The lesion is characterized by multiple histological features, starting from subepidermal heterophilic infiltration to necrobiotic-degenerative changes, which first affect the superficial epidermal layers and, as the lesion progresses from erosion to ulcer, involve all the epidermidis penetrating deep into the dermis (4). It causes pain, impedes perching and walking, and may limit access to food and water (5) and, if left untreated, FPD will compromise the internal tissues of the foot, such as the mesoderm, tendons and bones, causing osteomyelitis, synovitis, laminitis, and eventually death (6). The aetiology of FPD is multifactorial, involving genetic, nutritional and management-related causes (3). However, wet litter is identified as the main cause (7–9). Litter quality is of great importance for the welfare of broiler chickens as they generally spend their entire life in contact with it. Litter quality will affect the environmental condition of the birds by influencing dust levels, air humidity and ammonia levels, which, in turn, can lead to respiratory problems. Moreover, wet litter represents a major risk factor for contact dermatitis, having a direct influence on the skin condition of the birds (10). When stocking density is increased, litter quality worsens, leading to an increased incidence of FPD, but this relationship may not be so evident when the increased stocking density is compensated by improvements in management factors such as ventilation capacity (11). Litter materials with a high water-holding capacity, such as wood shaving, are believed to result in better litter quality than litter materials with poorer absorption capacity such as straw (10). Birds kept on chopped straw or wood shavings, both in winter and in summer seasons, exhibited a reduction of 35% in FPD compared to those kept on straw. Other bedding materials, such as peat moss or sawdust, have a high-water holding capacity but resulted in a dusty environment (12). Recently, attention has been directed towards the utilization of alternative bedding materials in commercial poultry houses, focusing on their efficacy in terms of availability and foot welfare (13). FPD not only affects welfare aspects, but also technical performance and carcass yield (14). Severe footpad lesions (FPL) are considered painful for birds (15), negatively impacting locomotion and causing a significant reduction in feed and water intake, resulting in a lower body weight gain and a higher feed conversion ratio (14). Furthermore, the presence of FPL is an important barrier to the sale of chicken feet, which have become an important commodity in the international market, representing part of the regular diet for people in some Asian countries (16), with China and Hong Kong representing the primary buyers of chicken feet in the world (17, 18).

Thus, we aimed to evaluate the efficacy of a bacterial compound, added to the litter, on growth performances, FPD and litter microbiological and chemical composition. Considering that in the last years non-invasive indicators are proposed to assess the suitability of farming methods and minimize the impact of stress on animal welfare (19–21), the application of open-source software to evaluate the extent of footpad lesions was also assessed.

## Material and methods

### Animals and experimental design

The trial was carried out in two poultry houses (C, control; T, treated), with a floor area of 2,150 m^2^ (length 119.45 × 18 m), located on a farm working in meat chicken production in the Marche Region, Italy. From a construction point of view, both buildings had the same equipment. The walls and the roof were made with 50 mm panels sandwiching internal polyurethane. The ventilation technology consisted of air-forced fans with a capacity of 45,000 m^3^/h, installed at the ends of the walls. Tunnel type, with darkened windows with automatic opening, for the heating air generators, with three burners and 10 radiant hoods, was adopted. Cooling panels were also installed. Before each new production cycle, the shed is completely cleaned, first mechanically then with water and a foaming agent based on quaternary ammonium salts. Finally, it is rinsed and disinfected, according to a standard hygienic protocol. Tap water and feed, whose chemical composition is reported in Table 1, were administered *ad libitum* by five lines of nipples each and four lines of feeders (7 cm/bird). Both the poultry houses were lighted with conventional light systems based on compact fluorescent lights, which assured a lighting program respecting the directive on the welfare of broilers in intensive breeding (1, 22). In both sheds, the litter was composed by wheat straw that was spread over the concrete pavement to a depth of 3 cm before the housing of one-day-old broiler chickens. After 2 weeks of farming, bales of wheat straw were distributed as environmental enrichment, to add to the litter 2 kg/m^2^ of straw after the dispersion carried out by the chickens themselves during the cycle. No other addition of fresh bedding occurred during the rest of the study. A total of 44,600 Ross308 chickens (initial mean body weight: 39 ± 3 g) were housed after hatching in each poultry house, at the same environmental conditions. House temperature was gradually reduced from 32 °C on day 1– 22 °C on day 20 and through the trial. Females (22,300) were housed in the first part of each shed (F), and males (22,300) in the second (M1) and third part (M2). The immunization protocol included Marek’s disease, infectious bronchitis and Newcastle disease vaccinations directly in incubator, with only a booster for infectious bronchitis in the farm. In each poultry house, 100 females and 100 male chickens were randomly weighed at arrival and then weekly, using a manual poultry scale (BAT1, VEIT Electronics, Moravany, Czech Republic). A bacterial bedding conditioner (EAZYBED DRY, Lallemand SAS, Blagnac, France) containing *Bacillus velezensis*, *Pediococcus acidilactici* and *Pediococcus pentosaceus* at 10^7^ CFU/g was applied in T: a pre-treatment (30 g/m^2^) was carried out on the floor before placing the litter and, from week 1 to the end of the cycle, the conditioner was applied weekly on the litter (90 g/m^2^). In C no treatment was applied. Females were slaughtered at 36 days, and from that day the males utilized the whole space until the end of the cycle (43 days).

**Table 1:** Chemical composition (% as is) and metabolizable energy calculated (ME) of the feeds administered to chickens in both poultry houses.

	Starter (0–10 d)	Grower I (10–21 d)	Grower II (22–36)	Finisher (37–42)
Crude protein	22.8	20	19.5	17.9
Lipids	5.5	6.6	8.2	7.6
Crude fibre	3.2	3.1	3.3	2.9
Ash	6.4	5.4	4.9	4.4
Lysine	1.48	1.29	1.26	1.13
Methionine	0.36	0.31	0.30	0.28
Calcium	0.94	0.77	0.66	0.59
Phosphorus	0.60	0.49	0.42	0.37
Sodium	0.16	0.16	0.16	0.15
ME (kcal/kg)	3,010	3,175	3,180	3,225

**Figure 1:**
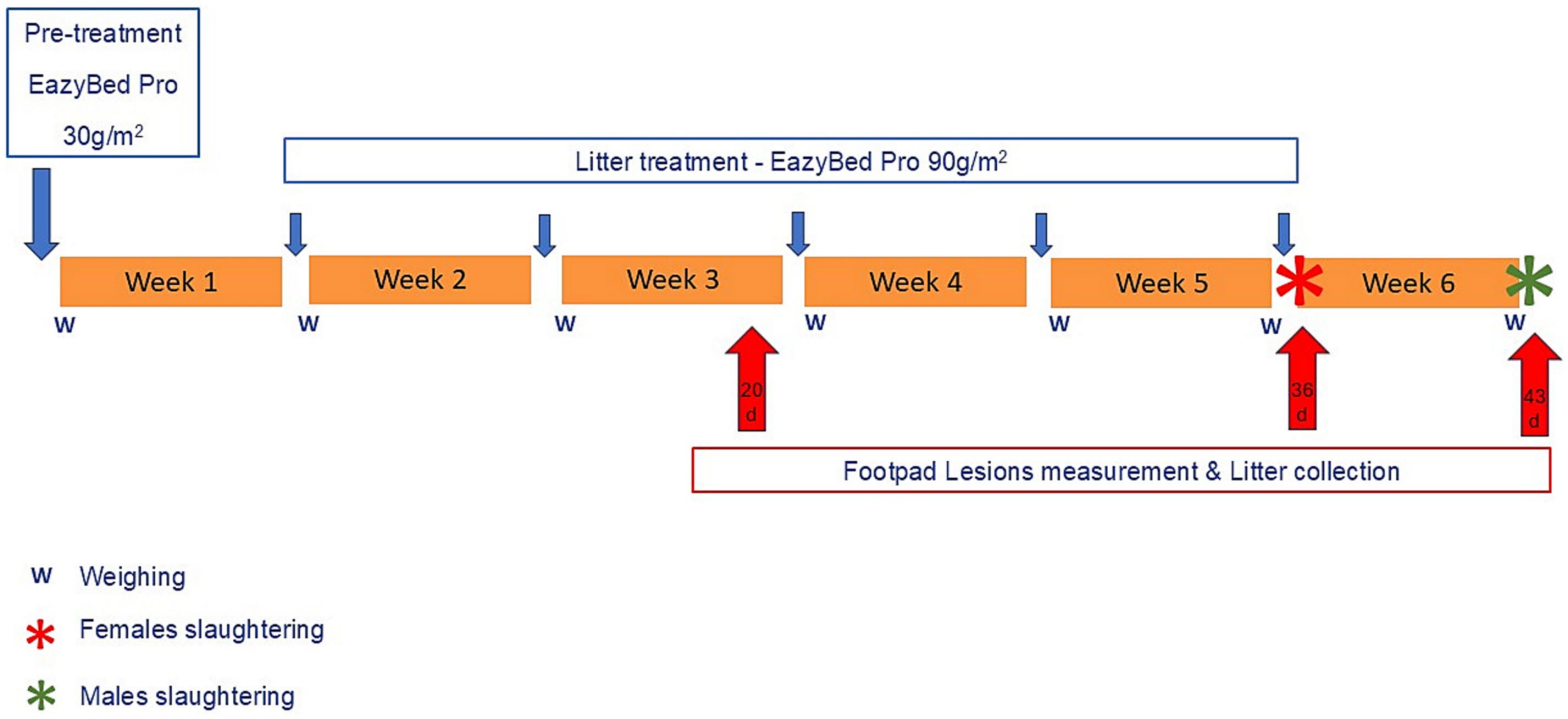
Timeline of the experimental trial.

The experimental design is described in Figure 1.

#### In-vivo footpad lesions measurement

In C and T, at 20d and before capture (36d and, only for males, 43 days), 30 birds living in each part of the poultry house were randomly selected and, from both pads, the perimeter shape of each lesion was recorded on transparent sheets. The perimeter shape of each wound area was collected by hand with a fine tip permanent marking pen on transparent sheets placed directly on the wound bed area on the plantar surface of the foot. Measurements of FPL areas in cm^2^ were obtained using the imitoMeasure application for mobile digital smart devices (Imito AG, Zürich, Switzerland, version 3.0.1). The app was used on a tablet (iPad Air, 4th generation, 10.9-inch screen, running operating system iPadOS 16.2, Apple Inc., Cupertino, California). The measurement procedure consisted of some fast phases. To obtain accurate measurements of the wound shapes a calibration was preliminarily performed. The special calibration marker (quick response [QR] code) was positioned on the transparent sheet where wound shapes were drawn, next to and in the same plane of the area of interest to be measured. A photograph was taken after recognition of the QR code by the imitoMeasure app., with the smart device positioned 20–30 cm away from and parallel to the transparent sheet. The area of interest was encircled on the tablet screen using a wireless digital pencil (Apple Pencil, 2nd generation, Apple Inc., Cupertino, California). When necessary, a fine-tuning of the selected shape was done by dragging the automatically created anchor points with the digital pencil. Once the appropriate wound shape was achieved, immediate measurement was obtained by the app and the area of each wound shape was collected (23). In case of multiple or bilateral injuries, the sum of the wound areas for each animal was considered.

### Litter bacteriological, mycological and chemical analyses

For bacteriological and mycological culture, litter was sampled collecting the entire layer, deep down to the cement, and pooling nine samples in each part [first (F), second (M1) and third (M2)] of the poultry house, at the same time points of FPL measurement (20d, 36d, 43d). The nine samples were obtained dividing each part in nine squares and sampling the litter at the centre of each square. The microbiological investigation involved the use of nutritive, selective and differential media for the isolation of aerobic, microaerophilic and anaerobic bacteria [Columbia Agar (Sheep blood 5%), Columbia CNA MOD. Agar (Sheep blood 5%), MacConkey Agar, Hektoen Enteric Agar, Liofilchem®, Roseto degli Abruzzi, Italy]. Selective and chromogenic media (Liofilchem®, Roseto degli Abruzzi, Italy) were used to detect *Staphylococcus aureus* (Chromatic Staph aureus Agar), *Pseudomonas aeruginosa* (Cetrimide Agar), *Bacillus cereus* (*B. cereus* Selective agar: PEMBA, Liofilchem®, Roseto degli Abruzzi, Italy), *Salmonella* spp., after pre-enrichment in Rappaport Vassiliadis broth, *Campylobacter* spp. (Campylobacter Agar-Sheep Blood 5%, Liofilchem®, Roseto degli Abruzzi, Italy), *Mycoplasma* spp., *Ureaplasma* spp. (Mycoplasma System Vet), *Clostridium* spp. (Clostridium S.P.S. Agar, Liofilchem®, Roseto degli Abruzzi, Italy), De Man–Rogosa–Sharpe agar (MRS) for Lactobacilli, bacteria following standard protocols (24). Plates were incubated for 24–48 h at 37 °C aerobically, anaerobically or in microaerophilic atmosphere (CampyGen and AnaGen, ThermoFisher, Waltham, MA, USA). For the detection of yeasts and fungi, Sabouraud Dextrose Agar with chloramphenicol (Liofilchem®, Roseto degli Abruzzi, Italy) was used and plates were incubated at 30 °C aerobically for at least 7 days.

The microorganisms were identified using MALDI-TOF MS (SOP Direct Transfer Procedure Revision.4; Bruker Microflex Lt®, Bruker Daltonics, Bermen, Germany). Mass spectra were processed using Flex Analysis (version 3.4; Bruker Daltonics, Bermen, Germany) and BioTyper software (version 3.1; Bruker Daltonics, Bermen, Germany). The row spectra obtained were compared with those present in the Biotyper database and log (score) ≥ 2.0 was considered.

To evaluate the total bacterial and fungal loads, 5 grams of litter were suspended with 50 mL of sterile saline solution (ThermoFisher, Waltham, MA, USA) for 30 min at room temperature and then vortexed for 30 s. After dilution at 1:10^6^ or 1:10^8^ or 1:10^10^ fold, aliquots of 100 μL were spread onto Plate Count Agar (Liofilchem®, Roseto degli Abruzzi, Italy) and Sabouraud Dextrose Agar with chloramphenicol (Liofilchem®, Roseto degli Abruzzi, Italy). In particular, to investigate a differentiated bacterial load, selected media used for qualitative analysis were employed.

For each plate, the number of colonies forming unit (CFU) was converted into number of microorganisms per gram of fresh litter. Each sample was examined in triplicate.

Chemical analyses of litter in the two poultry houses have been performed on the same samples to determine Total Kjeldahl Nitrogen, Ammoniacal Nitrogen, Dry Matter, Volatile Solids, pH in KCl. All parameters were analysed according to standard methods (25).

#### Histological analysis

At slaughtering, 12 legs from males and 12 from females, both C and T, were randomly selected for histological examination. FPL were collected, fixed in 10% neutral buffered formalin for 24 h, and routinely processed. Three-μm paraffin sections were placed on Superfrost Plus slides (Histoline, Milano, Italy). The slides were then dewaxed and stained with haematoxylin and eosin (H&E) for microscopic examination. Using a scoring system, several parameters were analysed: keratinization, epidermal layer structure, inflammation, leukocytes (heterophiles, macrophages, and lympho-plasmacytes), neoangiogenesis, dermal and hypodermal involvement (Table 2). In addition, to investigate the mechanism of lesion development, the number of apoptotic cells (DeadEnd™Colorimetric TUNEL System, Promega Italia Srl, Milano, Italy) and the expression of Hypoxia Inducible Factor (HIF-1α, dilution 1:200, MA1-516, Invitrogen, Waltham, MA, USA) were also assessed by immunohistochemistry. Tissues were counterstained with Mayer’s haematoxylin. For negative immunohistochemical controls, the primary antibodies were omitted. The absence of primary antibody did not result in immunoreactivity (26). During all the analysis, pathologist was blinded to the group allocation.

**Table 2:** Histological scores.

Score	Keratinization
0	Normal keratinization without signs of dyskerathosis and alteration of the *stratum corneum*
1	Slight signs of dyskeratosis with orthokeratosis
2	Dyskeratosis with orthokeratosis, and central erosion of sampled area
3	Dyskeratosis with orthokeratosis, and central ulcer of sampled area
4	Dyskeratosis with orthokeratosis, central ulcer with signs of hyperplasia in peripheral ulcerated area
5	Dyskeratosis with orthokeratosis, large ulcerative area without signs of reparation or hyperplasia in peripheral area of ulcer (phagedenic ulcer)

Score	Epidermis
0	Normal epidermal layer
1	Slight signs of achanthosis no infiltration of inflammatory cells
2	Achanthosis and mild infiltration of inflammatory cells, with superficial erosion
3	Achanthosis, spongiosis of some group of keratinocytes, moderate infiltration of inflammatory cells, central ulceration
4	Moderate achanthosis, spongiosis, severe infiltration of inflamm., ulceration with signs of peripheral hyperplasia
5	Severe achanthosis (caput medusa), spongiosis, severe infiltration of inflammatory cells, large ulceration without signs of peripheral hyperplasia/reparation

Score	Inflammation (general score, for HPF, 400X)
0	no/sporadic inflammatory cells for high-power field, 0–5 cells
1	few inflammatory cells, 5–10 cells
2	moderate number of inflammatory cells, 20–40 cells
3	high number of inflammatory cells, 50–100 cells
4	very high number of inflammatory cells, 100–200 cells
5	maximum number of inflammatory cells, 200 or more

Score	Etherophyls, Macrophages, and Lympho-plasmacytes (for HPF, 400X)
0	Absent, 0–19 cells
1	Mild, for 20–49 cells
2	Moderate, for 50–99 cells
3	Severe, for 100–200 cells or more

Score	Neoangiogenesis (for HPF, 400X)
0	No sign of neo-vascularization (no microvessels)
1	Mild—few newly formed microvessels, 1–3 microcapillaries
2	Moderate-moderate number/several microvessels, 3–5 microcapillaries
3	Abundant—abundant number—many microvessels, 5 microvessels and more

Score	Dermal involvement
0	Normal derma, without signs of inflammatory infiltration. No alteration of collagen and elastic fibres.
1	Mild inflammatory infiltration, mild edema, absence of micro-hemorrhages. No alteration of collagen and elastic fibres.
2	Moderate inflammation and edema, some micro-hemorrhages. No alteration of collagen and elastic fibres.
3	Moderate inflammation and edema, diffuse hemorrhagic phenomena. Slight alteration of collagen and elastic fibres disposition.
4	Severe inflammation, diffuse edema, diffuse hemorrhagic phenomena, Fragmentation of collagen and elastic fibres.
5	Severe inflammation, diffuse edema, hemorrhagic phenomena. Fragmentation of collagen fibres and elastic fibres, sequestration of necrotic material and signs of granulomatosis

Score	Hypodermal involvement
0	Absence of inflammatory infiltrate, absence of edema and hemorrhages, absence of fibrosis;
1	Absence of inflammatory infiltrate, presence of mild edema and microhemorrhages, absence of fibrosis
2	Mild inflammatory infiltrate, presence of edema and microhemorrhages, absence of fibrosis;
3	Presence of inflammatory infiltrate, severe edema and microhemorrhages, presence of fibrosis.

### Statistical analysis

The statistical analysis was based on univariate models for group comparisons. The cardinal variables were analysed for the assumption of normality of the data distribution with the Shapiro–Wilk test; they were summarized using the arithmetic mean and the standard error of mean (sem). The normally distributed cardinal variables were compared with the Student’s *t*-test or with one-way ANOVA (Analysis of Variance) and Holm–Sidak multiple comparison test. The ordinal variables were summarized using median and range. The ordinal variables and not-normally distributed cardinal variables were analysed via a nonparametric approach using Mann–Whitney test or Kruskal–Wallis test and Dunn’s multiple comparison test. Data deriving from categorical variables were evaluated with Fisher’s exact test. The significance level threshold was a *p*-value < 0.05. All data were analysed using GraphPad Prism 10 statistical software for MacOS, version 10.1.1-270 (GraphPad Software Inc., San Diego, CA, USA).

## Results

### Animals

At slaughtering time (36d), females averagely reached 1,528 ± 195 g in T and 1,562 ± 188 g in C whereas males completed the farming cycle after 43d weighing 2,696 ± 296 g in T and 2,737 ± 364 g in C. In both sexes, no significant differences were shown between the two poultry sheds.

#### *In-vivo* footpad lesions measurement

**Figure 2:**
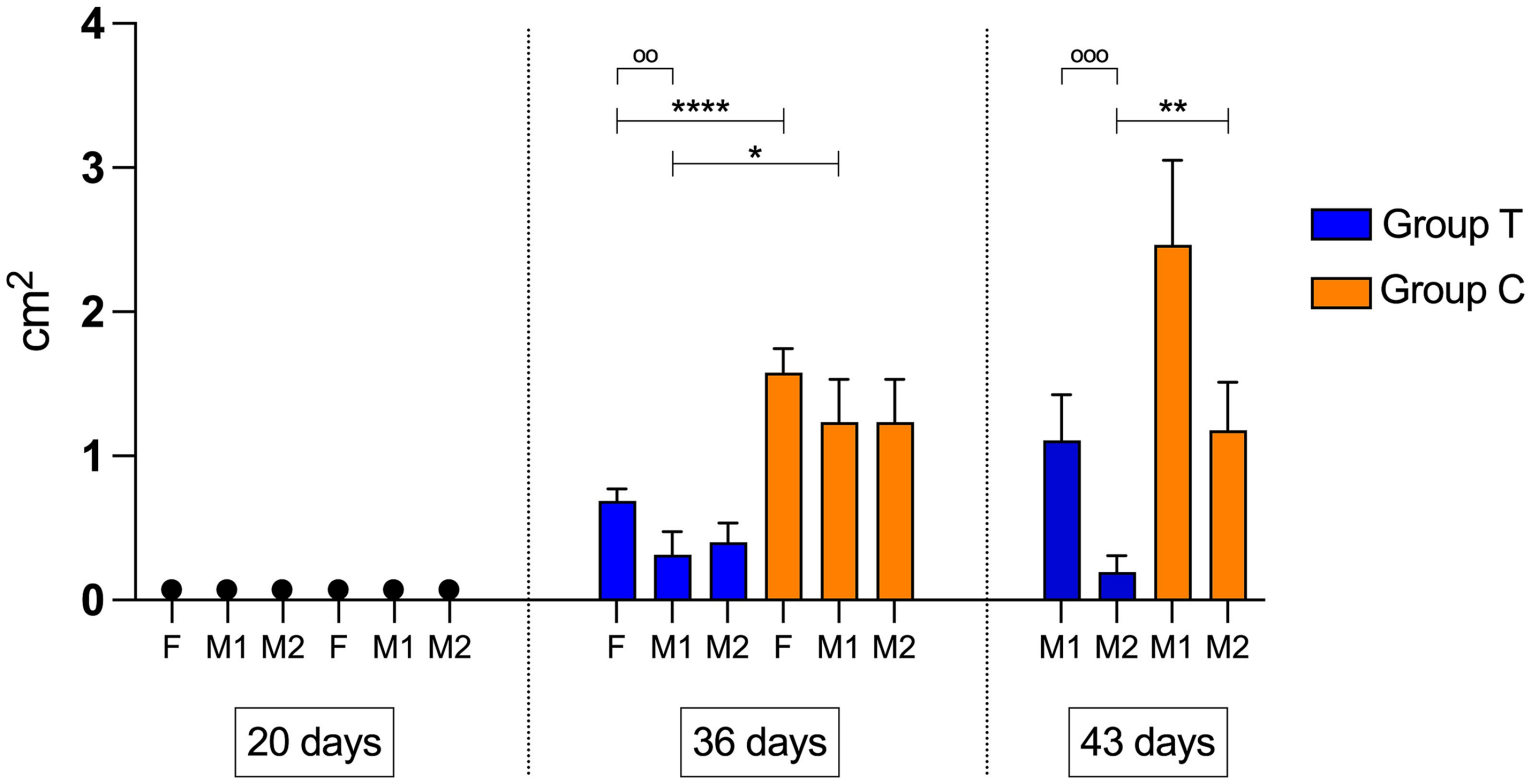
Mean values ± standard error of FPL areas in cm^2^ in groups (Group T: treated group; Group C: control group) and subgroups (F: females housed in the first part of the shed; M1: males housed in the second part of the shed; M2: males housed in the third part of the shed). Black dots indicate the lack of measurements due to the non-manifestation of the FPL. Asterisks indicate significant differences between groups. Circles indicate significant differences within each group; *p*-values, *: *p* < 0.05; **/°°: *p* < 0.01; °°°: *p* < 0.001; ****: *p* < 0.0001.

**Figure 3:**
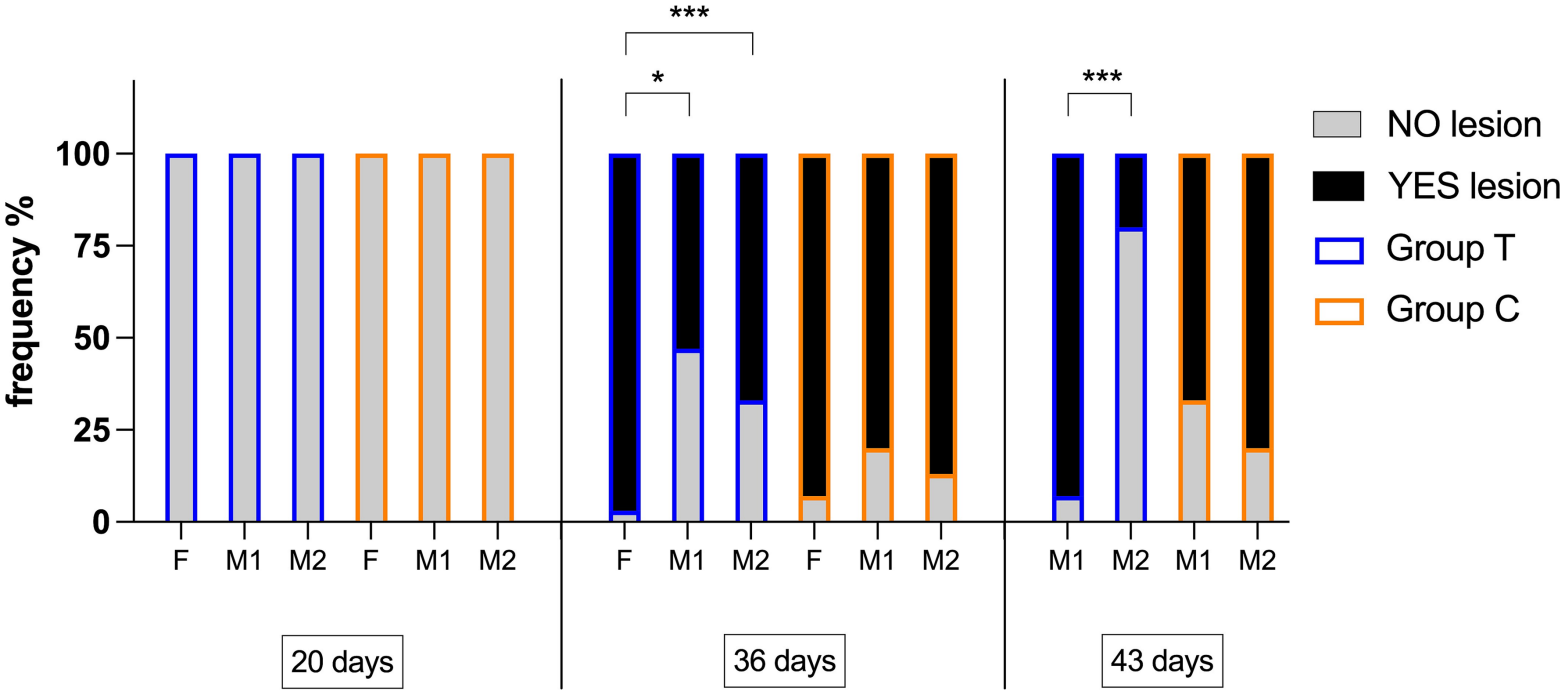
Stacked bars graph showing the percentage frequency of birds showing FPL during the study in groups (Group T: treated group; Group C: control group) and subgroups (F: females housed in the first part of the shed; M1: males housed in the second part of the shed; M2: males housed in the third part of the shed). Asterisks indicate significant differences. Circles indicate significant differences; *p*-values, *: *p* < 0.05; ***: *p* < 0.001.

At 20d, FPL were not observed in birds. At 36d, lesions measured in T were significantly lower than in C (*p* < 0.0001). At 43d, lesions measured in T were significantly lower than in C (*p* = 0.049). Considering the subgroups (F: females housed in the first part of the shed; M1: males housed in the second part of the shed; M2: males housed in the third part of the shed), significant differences were found in size of lesion (Figure 2) and frequency of appearance of the lesion (Figure 3).

#### Litter bacteriological, mycological and chemical analysis

In litter, 39 bacterial species and 5 fungal species were isolated (Table 3). In litter collected in T, Gram negative bacteria (*p* = 0.0015) and *Staphylococcus* spp. (*p* = 0.0386) significantly decreased (CFU/g) in respect to C, in particular in the second part of the shed (*p* = 0.0098 and *p* = 0.0131, respectively). Considering the single shed, no difference about bacterial loads was recorded between F, M1 and M2. Fungal flora does not express significant differences between sheds nor between parts of each shed.

**Table 3:** Bacterial and fungal species isolated from the litter during the trial.

Microorganisms MALDI-TOF MS	Poultry house C 20d			Poultry house T 20d			Poultry house C 36d			Poultry house T 36d			Poultry house C 43d			Poultry house T 43d		
Gram positive	F	M1	M2	F	M1	M2	F	M1	M2	F	M1	M2	F	M1	M2	F	M1	M2
*Aerococcus viridans*	X	X	X	X	X	X	X	X	X	X	X	X	X	X	X	X	X	X
*Bacillus pumilus*	X	X	X				X											
*Brevibacterium linens*													X	X	X	X	X	X
*Clostridium beijerinckii*														X		X	X	X
*Clostridium innocuum*													X					
*Clostridium novyi*	X	X	X	X		X												
*Clostridium tetani*	X	X	X	X		X												
*Corynebacterium amycolatum*							X	X	X	X	X	X						
*Corynebacterium stationis*	X	X	X	X	X	X	X	X	X	X	X	X				X	X	X
*Enterococcus faecalis*	X	X	X	X	X	X	X	X	X									
*Methylobacterium organophilum*													X			X	X	X
*Staphylococcus arlettae*	X	X	X	X	X	X	X	X	X	X	X	X	X					
*Staphylococcus condimenti*																X	X	X
*Staphylococcus conhii* ssp. *conhii*													X	X	X	X	X	X
*Staphylococcus conhii* ssp. *urealythicus*																X	X	X
*Staphylococcus lentus*							X	X	X	X	X	X						
*Staphylococcus lutrae*													X	X	X	X	X	X
*Streptococcus massiliensis*													X		X	X	X	X
*Streptobacillus moniliformis*																X	X	X
*Staphylococcus sciuri* ssp. *sciuri*													X			X	X	X
*Staphylococcus succinus* ssp. *succinus*													X	X	X	X	X	X
*Staphylococcus xylosus*	X	X	X	X	X	X												
Gram negative	F	M1	M2	F	M1	M2	F	M1	M1	F	M1	M2	F	M1	M2	F	M1	M2
*Aromatoleum aromaticum*													X	X	X	X	X	X
*Bacteroides fragilis*	X	X	X	X	X	X												
*Burkholderia anthina*	X	X	X	X	X	X												
*Escherichia coli*	X	X	X	X	X	X	X		X	X		X	X	X	X	X	X	X
*Fictibacillus macauensis*													X	X	X	X	X	X
*Klebsiella pneumoniae* ssp*. pneumoniae*				X														
*Lactobacillus amylovorus*													X	X	X	X	X	X
*Lactobacillus crispatus*														X				
*Lactobacillus kalixensis*													X	X	X			
*Lactobacillus paracasei* ssp. *paracasei*													X	X	X	X	X	X
*Lactobacillus plantarum*				X	X	X				X	X	X						
*Proteus mirabilis*	X	X	X		X	X					X			X				
*Pseudomonas corrugata*													X			X	X	X
*Pseudomonas libanensis*	X	X	X			X												
*Pseudomonas putida*																X	X	X
*Pseudomonas resinovorans*	X	X	X	X	X	X												
*Pseudomonas* sp.													X					
Fungi																		
*Diutina catenulata* (formerly *Candida catenulata*)					X									X				
*Diutina rugosa* (formerly *Candida rugosa*)					X	X							X	X	X	X	X	X
*Curvalaria clavata*											X							
*Penicillium digitatum*									X	X		X						
*Scopulariopsis brevicaulis*	X		X	X	X	X									X			

Results of chemical analyses of the litter, reported in Table 4, show a trend during the cycle with an increase of the nitrogen content during time. The dry matter decreases from day 20 to day 36 and then increases at day 43. The concentration of total nitrogen at day 36 and 43 is lower in T than in C but with a higher fraction of ammoniacal nitrogen. In fact, the ammoniacal nitrogen represents 9.1% of the total nitrogen in T while at day 36 it accounts for 5.7% in C and, respectively, the 4.6 and 3.1% at day 43. Conversely, the dry matter is slightly lower in T at both days 36 and 43.

**Table 4:** Chemical composition (wet basis) of the litter sampled during the study for the two groups.

Days	Groups	Total kjeldahl nitrogen mean ± SD (g/kg of raw material)	Ammoniacal nitrogen mean ± SD (g/kg of raw material)	Dry matter mean ± SD (g/kg of raw material)	Volatile solids (% of dry matter)	pH in KCl
20	T	18.93 ± 0.27	0.52 ± 0.02	74.3 ± 0.1	86.5	7.11
	C	18.56 ± 0.42	0.75 ± 0.08	71.1 ± 0.3	87.0	6.89
36	T	23.55 ± 0.14	2.15 ± 0.03	56.3 ± 0.3	85.8	6.83
	C	26.84 ± 0.33	1.52 ± 0.03	67.5 ± 0.4	84.9	6.98
43	T	28.10 ± 0.06	1.28 ± 0.02	78.9 ± 0.3	85.0	7.46
	C	32.40 ± 0.44	0.99 ± 0.02	82.5 ± 0.2	83.8	7.30

SD, standard deviation; T, treated group, C, control group.

#### Histological analysis

**Figure 4:**
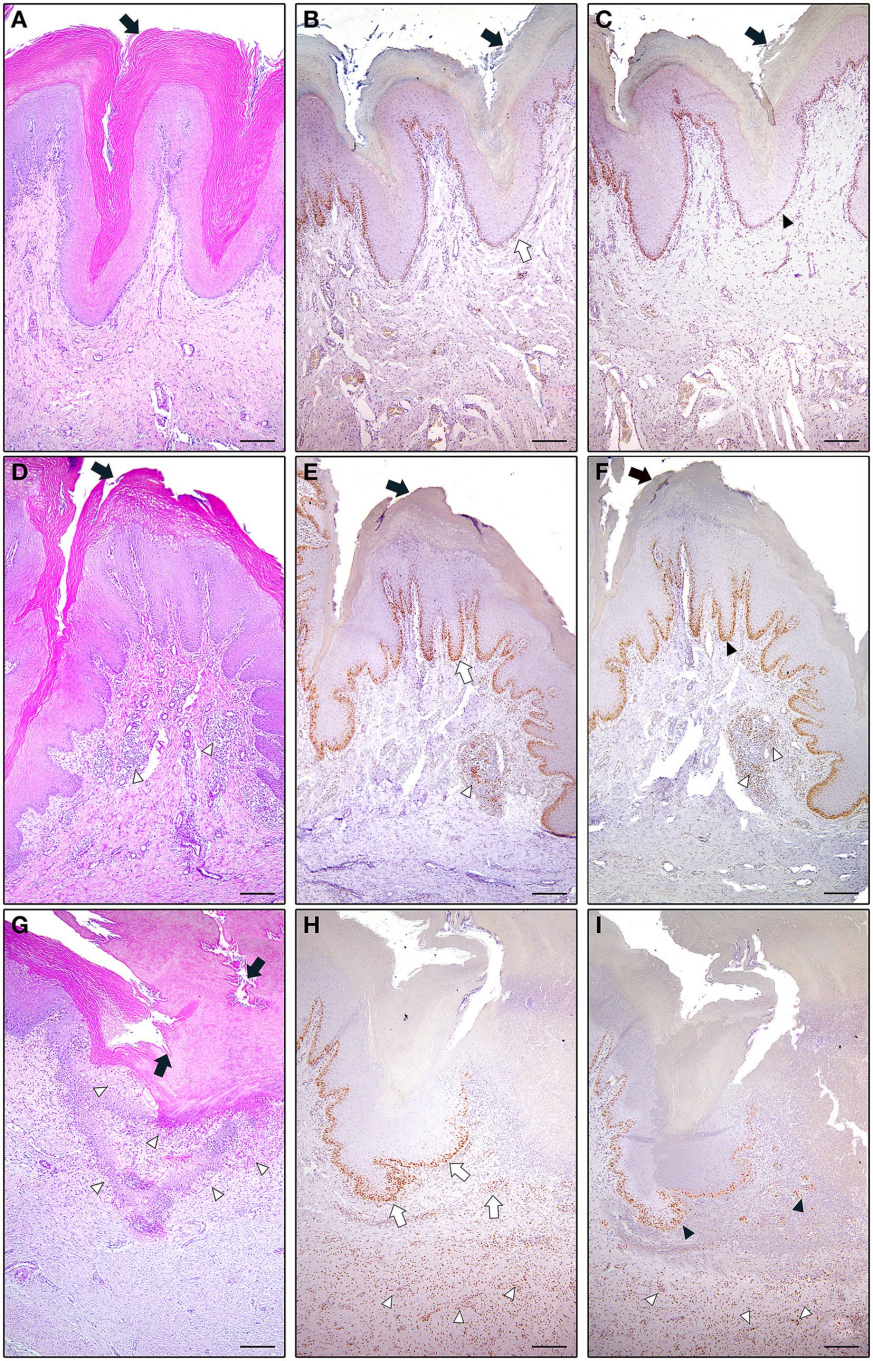
Footpad dermatitis. Histological aspects of footpad lesions in broilers maintained on different types of litters. **(A–C)** Footpad aspect of female broiler kept in shed T. Note the hypertrophic but compact and non-disintegrated keratin layer (**A**, arrow), with the presence of a normal and non-hyperplastic basal layer. Moderate to poor inflammatory infiltrate and continuous level of apoptosis of the cells of the basal layer (**B**, open arrow), with numerous T.U.N.E.L positive nuclei. A moderate expression is noted, by the same TUNEL+ cells, also of the HIF factor (**C**, arrowhead). **(D-F)** Footpad aspect of male broiler kept in shed T. Hyperkeratosis and separation of the keratin layers was seen in these birds (**D**, arrow). Hyperkeratosis refers to a rapid turnover of keratinocytes that are undergoing apoptosis to produce keratin, resulting in a thickened layer of underdeveloped keratin. Lymphocyte, granulocyte, and lymph follicle populations increased in the dermis under the hyperplastic-acanthotic basal layer (**D**, open arrowhead) adjacent to the lesions. Note an increased number of apoptotic/TUNEL+ cells at level of basal layer (**E**, open arrow), and in some inflammatory infiltrates (**E**, open arrowhead). In these broilers an increased number of HIF + cells is observed both at the basal layer (**F**, arrowhead), as in many inflammatory cells (**F**, open arrowheads). **(G–I)** footpad aspect of male broiler kept in shed C. Hyperkeratosis and severe ulceration of the footpad (**G**, arrows), with complete destruction of the keratin and epidermal layer in the centre of the lesion, with necrotic tissue exposed and a mass of heterophils (**G**, arrowheads). In these lesions, heterophils are also in the germ layer and defects in keratin formation are observed. Heterophils were also found in the dermis, subepidermis, and epidermis along with basophilic cells in the stratum corneum of these lesions. Vacuoles containing heterophils have been identifies within the epidermis and within blood vessels of the footpad (**G**, arrowheads). In **H**, note a large amount of TUNEL+ basal (open arrows), and inflammatory (open arrowheads) cells. In the area of severe lesion, there was acute inflammation with a denser cellular infiltration and a thickening of the stratum corneum, which were referred to as horned pegs. In these areas the expression of HIF antigen assumes a strong intensity in epithelial (**I**, arrowheads) and in inflammatory cells (**I**, open arrowheads). The epidermis is more eroded, and the dermis is filled with inflammatory cells, congestion and dilation of blood vessels. Scale bar = 500 μm.

T-chickens showed a total histological score significantly lower than C-birds (*p* = 0.0002), especially in males (*p* < 0.0001). Considering the single parameters, several differences were noted, except for the heterophiles count (*p* = 0.251). Treatment significantly reduced inflammation (*p* < 0.0001) and hypodermal involvement (*p* = 0.004), both in females and males. Only in males, keratinization (*p* = 0.018), epidermal layer structure (*p* = 0.012), leukocytes [macrophages (*p* = 0.016) and lympho-plasmacytes (*p* = 0.021)], neoangiogenesis (*p* = 0.027), dermal involvement of the lesions (*p* = 0.0004), were significantly reduced. HIF and TUNEL positive cells counts resulted significantly reduced in chickens farmed in T (*p* < 0.0001) both for females (*p* < 0.0001) and males (*p* < 0.0001). Figure 4 shows the histological aspects of footpad lesions in broilers in C and T.

## Discussion

In this study, the final average body weight exhibited satisfactory results without significant differences between broiler chickens raised on treated litter and control litter, illustrating thus the absence of any adverse effect of the bacterial compound. This result agrees with other studies in which antiseptic treatment was applied in litter (27) or chemical compound, such as sodium bisulphate, was added (28). Differently, previous studies noted that litter amendment, in addition to improving foot welfare, favoured better growth performances (29). This discrepancy can be attributed to the absence of deep ulcers in the foot lesions observed in poultry in C, which are known to cause pain that can impair movement and, consequently, reduce appetite.

The development of footpad lesions is multifactorial (30), and certainly bedding condition is a major contributor to ulcer development (31). In this trial, we observed a significant reduction of FPL in poultry living in the shed where a bacterial compound was added to the litter. Several methods are used to evaluate these lesions, but the comparison of different studies is compromised by the fact that scoring systems differ in respect to scoring criteria and scale (32). Some scoring systems are primarily based on the 2-dimensional size of lesions, as used in the United Kingdom (33, 34), while others in principle discriminate on 3-dimension, evaluating both size and depth of lesions as proposed in France (15, 35), Sweden (36), and Denmark (32). We tested the usefulness of imitoMeasure application, a device used to measure the size of wound in medicine (37), to objectify the obtained values. Even under field condition, the use of the application seems to be reliable, although more time-consuming than a visual scale. Indeed, the accuracy of the evaluation obtained in cm^2^ allows us to suggest the use of this method in experimental evaluations, when the accuracy of the data becomes more important than the speed of the evaluation.

The action of the compound added on the litter is also confirmed by the significant reduction of Gram-negative bacteria and *Staphylococcus* spp. count. These bacteria are often associated to FPL and chronic abscess in broilers and egg layers (31, 38). In particular, *Staphylococcus* spp., a ubiquitous Gram-positive bacterium, is present in high concentrations in the dust of poultry houses, animal feed, and gut contents and even on the skin of nonclinical animals (39, 40). When the skin barriers have been compromised, these bacteria can invade the mesoderm and proliferate, inducing inflammation (38) that can ultimately result in systemic disease (41).

Although it is not possible to perform a statistical analysis, the litter nitrogen contents indicate a possible effect of the bedding conditioner with a higher fraction of ammoniacal nitrogen content that might suggest a reduced ammonia emissions in T. The higher total nitrogen content in the litter in T can be explained by the different humidity content of the litter and the values calculated on dry matter basis are similar for T and C.

Histological analysis confirmed the positive effect of the bacterial compound applied on the litter, with a total score significantly lower in T than in C, indicating a reduced severity of the lesions. The number of cells with a positive immunostaining for TUNEL and HIF-1 permits to hypothesize a relation between the hypoxic condition of the wound and the percentage of apoptosis. Indeed, a key regulator of the hypoxia response is hypoxia-inducible factor 1 (HIF-1), which has been shown to induce apoptosis or, on the contrary, to prevent cell death and even stimulate cell proliferation under different conditions and stimuli (42). In the present study, we observed that the number of apoptotic/TUNEL positive and HIF-1 positive cells was higher in both the basal layer of the epidermis and in the inflammatory infiltrate of the area surrounding the lesion. This was particularly evident in birds with a higher histological score, indicating a greater severity of the lesion. These data support the hypothesis that, following the development of FPL, the condition worsens as a result of hypoxia-induced apoptosis mechanism. Hypoxia is in turn due to a direct compression of the foot region, which also correlates significantly with the weight of the animal. This detection explains the worst lesions in males, whose average weight is higher than in females. The positive function of the bacterial compound spread on the litter was supported by both histological and immunohistochemical evaluations. Indeed, a reduction in FPL size was observed in T along with a decrease in the severity of the lesion, in terms of reduced inflammation and involvement of the epidermis, dermis and hypodermis. These results suggest that concomitant factors may lead to a reduction in the bioavailability of oxygen at the footpad level and therefore lesions may be firstly induced by poor litter condition but, afterwards, the activation of the HIF gene and the increased induction of apoptosis may favour the expansion and aggravation of the lesion, preventing structural regeneration.

As litter represents the main solid leftover in broiler industry (43), in non-European countries it is possible to use the litter for multiple production cycles. The presence of residual bacteria is critical in assessing the microbiological risk of the recycling process of litter (44). In case of reuse of the litter, a bacterial bedding condition could indeed improve the microbiological quality of the litter itself.

## Conclusion

In our study, in both poultry sheds, chickens reached satisfactory mean body weights according to the expected performance of the strain and the use of a bacterial litter conditioner had a positive effect on the extent and severity of FPL, improving broilers welfare. Furthermore, future studies will be necessary to evaluate the use of the tested bacterial bedding conditioner on a large scale and in other avian species like turkey and laying hen characterized by a long production cycle, notably in case of litter reuse.

## Data Availability

The original contributions presented in the study are included in the article/supplementary material, further inquiries can be directed to the corresponding author.

## References

[1] Council directive 2007/43/EC of 28 June 2007 laying down minimum rules for the protection of chickens kept for meat production. Available online at: https://eur-lex.europa.eu/legal-content/EN/TXT/PDF/?uri=CELEX:32007L0043 (Accessed May 12, 2025).

[2] Report from the commission to the European Parliament and the council on the application of directive 2007/43/EC and its influence on the welfare of chickens kept for meat production, as well as the development of welfare indicators. (2018). Available online at: https://eur-lex.europa.eu/legal-content/EN/TXT/PDF/?uri=CELEX:52018DC0181 (Accessed May 12, 2025).

[3] Shepherd EM Fairchild BD. Footpad dermatitis in poultry. Poult Sci. (2010) 89:2043–51. doi: 10.3382/ps.2010-00770, PMID: 20852093

[4] Dinev I Denev S Vashin I Kanakov D Rusenova N. Pathomorphological investigations on the prevalence of contact dermatitis lesions in broiler chickens. J Appl Anim Res. (2019) 47:129–34. doi: 10.1080/09712119.2019.1584105

[5] Hester PY. The role of environment and management on leg abnormalities in meat-type fowl. Poult Sci. (1994) 73:904–15. doi: 10.3382/ps.0730904, PMID: 8072936

[6] McNamee PT Smyth JA. Bacterial chondronecrossis with osteomyelitis (femoral head) of broiler chicken: a review. Avian Pathol. (2000) 29:253–70. doi: 10.1080/030794500750047243 19184815

[7] Martland MF. Wet litter as a cause of plantar pododermatitis, leading to foot ulceration and lameness in fattening turkeys. Avian Pathol. (1984) 13:241–52. doi: 10.1080/03079458408418528, PMID: 18766841

[8] Mayne RK. A review of the aetiology and possible causative factors of foot pad dermatitis in growing turkeys and broilers. Worlds Poult Sci J. (2005) 61:256–67. doi: 10.1079/WPS200458

[9] Hinz K Stracke J Schättler JK Spindler B Kemper N. Foot pad health and growth performance in broiler chickens as affected by supplemental charcoal and fermented herb extract (FKE): an on-farm study. Eur Poult Sci. (2019) 83:266. doi: 10.1399/eps.2019.266

[10] The welfare of chickens kept for meat production (broilers). Report of the scientific committee in animal health and animal welfare. Brussels, Belgium: European Commission, Health and Consumer Protection Directorate General (2000).

[11] Berg CC. Foot-pad dermatitis in broilers and turkeys. Acta Univ Agric Sueciae Vet. (1998) 36:7–43.

[12] Toppel K Kaufmann F Schön H Gauly M Andersson R. Effect of pH-lowering litter amendment on animal-based welfare indicators and litter quality in a European commercial broiler husbandry. Poult Sci. (2019) 98:1181–9. doi: 10.3382/ps/pey489, PMID: 30325450

[13] Diarra S Lameta S Amosa F Anand S. Alternative bedding materials for poultry: availability, efficacy, and major constraints. Front Vet Sci. (2021) 8:669504. doi: 10.3389/fvets.2021.669504, PMID: 34485425 PMC8416037

[14] De Jong IC Gunnink H Van Harn J. Wet litter not only induces footpad dermatitis but also reduces overall welfare, technical performance, and carcass yield in broiler chickens. J Appl Poult Res. (2014) 23:51–8. doi: 10.3382/japr.2013-00803

[15] Michel V Prampart E Mirabito L Allain V Arnould C Huonnic D . Histologically-validated footpad dermatitis scoring system for use in chicken processing plants. Br Poult Sci. (2012) 53:275–81. doi: 10.1080/00071668.2012.695336 22978583

[16] Giron TV Vieira BS Viott AM Pozza MSS Castilha LD Reis IN . Mechanical removal (epidermal scarification) of pododermatitis injuries reduces the presence of both inflammatory tissue and its associated microbiota in broiler feet. Poult Sci. (2019) 98:1455–60. doi: 10.3382/ps/pey497, PMID: 30325460

[17] United States Department of Agriculture (USDA). (2015). International egg and poultry review. Livestock, Poultry and Grain Market News. 18: 16. Available online at: https://www.ams.usda.gov/mnreports/pywintlpoultryandegg.pdf (Accessed May 13, 2025)

[18] United States Department of Agriculture (USDA). (2006). Foreign Agricultural Service. Challenges to increasing US sales of chicken paws to China. Available online at: https://apps.fas.usda.gov/newgainapi/api/Report/DownloadReportByFileName?fileName=Challenges+to+Increasing+U.S.+Sales+of+Chicken+Paws+to+China_Beijing_China-+Peoples+Republic+of_06-15-2007.pdf (Accessed May 13, 2025)

[19] Galosi L Todini L Menchetti L Carbajal A Palme R Ruggiero N . Effect of a broiler-specific light spectrum on growth performance and adrenocortical activity in chickens: a pilot study on a commercial farm. Vet Sci. (2024) 11:618. doi: 10.3390/vetsci11120618, PMID: 39728958 PMC11680236

[20] Kjaer JB Glawatz H Scholz B Rettenbacher S Tauson R. Reducing stress during welfare inspection: validation of a non-intrusive version of the LayWel plumage scoring system for laying hens. Br Poult Sci. (2011) 52:149–54. doi: 10.1080/00071668.2011.554799, PMID: 21491236

[21] Stewart M Webster JR Schaefer AL Cook NJ Scott SL. Infrared thermography as a non-invasive tool to study animal welfare. Anim Welf. (2005) 14:319–25. doi: 10.1017/S096272860002964X

[22] Italian Legislative Decree 27 September 2010, n. 181. Implementation of directive 2007/43/EC establishing minimum standards for the protection of chickens reared for meat production. [Decreto Legislativo 27 settembre 2010, n. 181. Attuazione della direttiva 2007/43/CE che stabilisce norme minime per la protezione di polli allevati per la produzione di carne]. Available online at: https://www.normattiva.it/uri-res/N2Ls?urn:nir:stato:decreto.legislativo:2010;181 (Accessed May 17, 2025).

[23] Tambella AM Galosi M Angorini A Dini F Palumbo Piccionello A Di Bella C . Advances in noncontact measurement of wound area using an application for smart mobile devices. Adv Skin Wound Care. (2025). in print) 38:360–6. doi: 10.1097/ASW.0000000000000296, PMID: 40341457

[24] NHS - United Kingdom Standard Units, Microbiology Services. (2014). Public health, England. Available online at: https://www.rcpath.org/profession/publications/standards-for-microbiology-investigations.html (Accessed February 3, 2025).

[25] APHA. Standard methods for the examination of water and waste water. 22nd ed. Washington DC: American Public Health Association, American Water Works Association, Water Environment Federation (2012).

[26] Lorenzo-Betancor O Galosi L Bonfili L Eleuteri AM Cecarini V Verin R . Homozygous CADPS2 mutations cause neurodegenerative disease with Lewy body-like pathology in parrots. Mov Disord. (2022) 37:2345–54. doi: 10.1002/mds.29211, PMID: 36086934 PMC9772200

[27] Freeman N Tuyttens FAM Johnson A Marshall V Garmyn A Jacobs L. Remedying contact dermatitis in broiler chickens with novel flooring treatments. Animals. (2020) 10:1761. doi: 10.3390/ani10101761, PMID: 32998380 PMC7599451

[28] Nagaraj M Wilson CAP Saenmahayak B Hess JB Bilgili SF. Efficacy of a litter amendment to reduce pododermatitis in broiler chickens. J Appl Poult Res. (2007) 16:255–61. doi: 10.1093/japr/16.2.255

[29] Sahoo SP Kaur D Sethi APS Sharma A Chandra M Chandrahas. Effect of chemically amended litter on litter quality and broiler performance in winter. J Appl Anim Res. (2017) 45:533–7. doi: 10.1080/09712119.2016.1150846

[30] Kittelsen KE Vasdal G Thøfner I Tahamtani F. A walk through the broiler breeder life: how do footpad dermatitis and gait scores develop from rearing to slaughter? Avian Pathol. (2024) 53:164–73. doi: 10.1080/03079457.2024.2304005, PMID: 38193215

[31] Heidemann Olsen R Christensen H Kabell S Bisgaard M. Characterization of prevalent bacterial pathogens associated with pododermatitis in table egg layers. Avian Pathol. (2018) 47:281–5. doi: 10.1080/03079457.2018.1440066, PMID: 29517269

[32] Lund VP Nielsen LR Oliveira ARS Christensen JP. Evaluation of the Danish footpad lesion surveillance in conventional and organic broilers: misclassification of scoring. Poult Sci. (2017) 96:2018–28. doi: 10.3382/ps/pex024, PMID: 28204752

[33] Martland MF. Ulcerative dermatitis dm broiler chickens: the effects of wet litter. Avian Pathol. (1985) 14:353–64. doi: 10.1080/03079458508436237, PMID: 18766928

[34] Pagazaurtundua A Warriss PD. Measurements of footpad dermatitis in broiler chickens at processing plants. Vet Rec. (2006) 158:679–82. doi: 10.1136/vr.158.20.679, PMID: 16714430

[35] Allain V Mirabito L Arnould C Colas M Le Bouquin S Lupo C . Skin lesions in broiler chickens measured at the slaughterhouse: relationships between lesions and between their prevalence and rearing factors. Br Poult Sci. (2009) 50:407–17. doi: 10.1080/00071660903110901, PMID: 19735009

[36] Ekstrand C Algers B Svedberg J. Rearing conditions and foot-pad dermatitis in Swedish broiler chickens. Prev Vet Med. (1997) 31:167–74. doi: 10.1016/S0167-5877(96)01145-2, PMID: 9234440

[37] Gupta K Chittoria RK Mohan PB Chakiath JA Nepali S. Assessment of electric burns wound dimensions using a smartphone-based application-Imitomeasure in wound assessment. Clin Trials Case Stud. (2024) 3, 1–3. doi: 10.31579/2835-835X/068

[38] Wilcox CS Patterson J Cheng HW. Use of thermography to screen for subclinical bumblefoot in poultry. Poult Sci. (2009) 88:1176–80. doi: 10.3382/ps.2008-00446, PMID: 19439627

[39] Cotter PF Taylor RL Jr. *Staphylococcus aureus* carriage in commercial layers. Poult Sci. (1987) 66:86.

[40] Zhu XY Wu CC Hester PY. Induction of the delayed footpad and wattle reaction to killed *Staphylococcus aureus* in chickens. Poult Sci. (1999) 78:346–52. doi: 10.1093/ps/78.3.346, PMID: 10090260

[41] Thøfner ICN Poulsen LL Bisgaard M Christensen H Olsen RH Christensen JP. Correlation between footpad lesions and systemic bacterial infections in broiler breeders. Vet Res. (2019) 50:38–5. doi: 10.1186/s13567-019-0657-8, PMID: 31118094 PMC6532141

[42] Greijer AE Van der Wall E. The role of hypoxia inducible factor 1 (HIF-1) in hypoxia-induced apoptosis. J Clin Pathol. (2004) 57:1009–14. doi: 10.1136/jcp.2003.015032, PMID: 15452150 PMC1770458

[43] Coufal CD Chavez C Niemeyer PR Carey JB. Measurement of broiler litter production rates and nutrient content using recycled litter. Poult Sci. (2006) 85:398–403. doi: 10.1093/ps/85.3.398, PMID: 16553266

[44] Vaz CSL Voss-Rech D De Avila VS Coldebella A Silva VS. Interventions to reduce the bacterial load in recycled broiler litter. Poult Sci. (2017) 96:2587–94. doi: 10.3382/ps/pex063, PMID: 28371809

